# Prevalence and associated factors of postdural puncture headache in cesarean section patients following spinal anesthesia in a public hospital in Harar, Eastern Ethiopia

**DOI:** 10.1371/journal.pgph.0006581

**Published:** 2026-06-02

**Authors:** Gizachew Tilaye, Alemayehu Tesfaye, Awoke Getiye, Birhanu Shegene, Mengistu Chalie, Abebe Tolera

**Affiliations:** 1 School of Medicine, College of Health and Medical Sciences, Haramaya University, Harar, Ethiopia; 2 School of Public Health, College of Health and Medical Sciences, Haramaya University, Harar, Ethiopia; University of Global Health Equity, RWANDA

## Abstract

Post-dural puncture headache is becoming a major health problem around the globe. Even though it becomes the reason for increased maternal morbidity, there is still a great scarcity of research in Eastern Ethiopia. This study aimed to assess the prevalence and associated factors of post-dural puncture headache in patients undergoing cesarean sections following spinal anesthesia at Hiwot Fana Specialized University Hospital. An institution-based cross-sectional study was conducted among 291 patients from November 20/2024, to January 20/2025. An adjusted odds ratio presented the strength of association with a 95% confidence interval, and statistical significance was declared at a *p*-value less than 0.05. The overall prevalence of post-dural puncture headache in cesarean section patients was 39%. Previous history of spinal anesthesia (AOR = 2.05; 95% CI: 1.16-3.65, *p* = 0.014), previous history of post-dural puncture headache (AOR = 1.87; 95% CI: 1.08-3.25, *p* = 0.025), use of large spinal needle size (AOR = 2.25; 95% CI: 1.22-4.14, *p* = 0.009), and patients who had emergency cesarean sections (AOR = 2.72; 95% CI: 1.28-5.80, *p* = 0.010) have shown statistically significant associations with post-dural puncture headache. The prevalence of post-dural puncture headache in this study was higher than in most other studies. Factors such as previous history of spinal anesthesia, previous history of post-dural puncture headache, patients who received spinal anesthesia using bigger spinal needles, and respondents who had emergency cesarean sections were found to be significantly associated with post-dural puncture headache. The findings of this study suggest that the healthcare providers should pay special attention to emergency cesarean deliveries for women who have a previous history of spinal anesthesia and post-dural puncture headache, as well as providing smaller-gauge spinal needles and pencil-point needles, would be enormous in reducing post-dural puncture headache in patients undergoing cesarean sections.

## Introduction

Postdural puncture headache (PDPH) is becoming a major health problem around the globe and continues to be a significant cause of morbidity [1]. PDPH can result in longer hospital stays and increased healthcare costs, as well as dissatisfaction among women [2]. A prolonged or severe PDPH may lead to serious complications, including cerebral venous thrombosis, bacterial meningitis, hypopituitarism, seizures, herniation, coma, and even death [3]. Spinal anesthesia (SA) is currently used for cesarean sections all over the world; reports from various studies indicate that the percentage of SA used in the United Kingdom is 89.2% [4], 95.2% in Botswana [5], and 53.5% in Addis Ababa, Ethiopia [6]. One potential complication of SA is PDPH [7]. As mentioned in the diagnostic standards set by the International Headache Society (IHS), the headache can occur up to the fifth day following the puncture. It may resolve on its own within a week, or as early as 48 hours after an epidural blood patch, accompanied by neck stiffness, tinnitus, hypoacusia (partial loss of hearing), photophobia, and nausea [8].

PDPH is believed to be a result of a persistent cerebrospinal fluid (CSF) leak that surpasses the rate of CSF production following the puncture [9]. A meta-analysis revealed a pooled prevalence of PDPH of 23.47% (range, 10.53%-36.42%) [10]. The prevalence of PDPH was found to be 6.3% in a study done at King Abdullah University Hospital in Jordan among women who had cesarean deliveries [11]. A prospective study conducted at Aminu Kano Teaching Hospital in Kano, Nigeria, found that the overall prevalence of PDPH among pregnant women who had a cesarean section was 15.8% [12]. According to a study conducted in Gondar, Ethiopia, 38.8% of patients who had SA experienced PDPH [13].

It is thought that pregnant women have a higher risk of developing PDPH because high estrogen levels in women might change the tone of the cerebral arteries, which in turn can exacerbate the vascular distension response to CSF hypotension [14]. Being female, young age, and having a lean body weight are the risk factors for developing PDPH after SA [15]. Performing SA in the sitting position [16], when the bevel of the spinal needle is inserted perpendicularly to the longitudinal fibers of the dura [11,17], and repeated dural puncture attempts more than two [18] result in a higher risk of PDPH. The prevalence of PDPH is significantly higher when 25 G needles are used compared to 27 G needles [17]. The use of a narrow-gauge, pencil-tipped, non-cutting spinal needle is the most crucial factor in preventing PDPH [19,20].

Conservative therapy, such as bed rest, hydration, and caffeine, is commonly used as a preventative measures and a treatment for this condition; however, data are not very clear on the benefits of extensive hydration and consistent bed rest [14]. Adrenocorticotropic hormone, epidural morphine, and intravenous aminophylline may lower the prevalence of PDPH, although further research is required to confirm their effectiveness [21]. A study [22] revealed that the common recommendations in textbooks and review articles for using caffeine to prevent and treat post-dural puncture headache (PDPH) may be unjustified and lack sufficient pharmacological and clinical evidence. A randomized clinical trial revealed that the administration of 500 mg acetaminophen with 65 mg caffeine decreased the chance of PDPH development by 70% in the first 72 hours after surgery, and participants reported significantly milder headaches and higher overall satisfaction [23].

Few studies in Ethiopia examine the prevalence of PDPH and its associated factors in cesarean section patients who receive SA [13,18], and there is limited evidence on PDPH in the area of interest. Therefore, this study aimed to assess the incidence and associated factors of PDPH in cesarean section patients following SA at Hiwot Fana Comprehensive Specialized University Hospital (HFCSUH) in Harar, Eastern Ethiopia.

## Methods

### Ethics statement

An ethical clearance was obtained from the Institutional Health Research Ethics Review Committee (IHRERC) of Haramaya University College of Health and Medical Sciences with reference number (Ref. No IHRERC/284/2024), and a support letter was submitted to HFCSUH, where the study was conducted. Informed, voluntary, written, and signed consent was obtained from the head of the hospital and study participants. All study participants were informed about the purpose of the study. The participants’ right to self-determination and autonomy was respected. The information provided by participants was kept confidential by the principal investigator, and no information identifies the participants specifically. The findings of the study were general for the whole study population and did not reflect the particular participant.

### Study setting and design

An institution-based cross-sectional study design was conducted at HFCSUH, which is located in Harari Regional State, Eastern Ethiopia. Harar is the capital city of the Harari regional state, which is 532 km east of Addis Ababa, the capital city of Ethiopia. According to the Central Statistical Agency population projection by 2022, the region had an estimated population of 276,431 [24]. The region has two public hospitals (HFCSUH and Jugale General Hospital), 8 health centers, and 28 health posts. HFCSUH is a teaching hospital of Haramaya University with a total of 210 beds and more than 250 health professionals working in this hospital. According to the information obtained from the Hospital’s Health Management Information System or obstetric and maternity ward registry, an average of 180 cesarean sections is performed per month. The study was conducted from November 20/2024-January 20/2025, in Eastern Ethiopia.

### Populations and sampling

**Fig 1:**
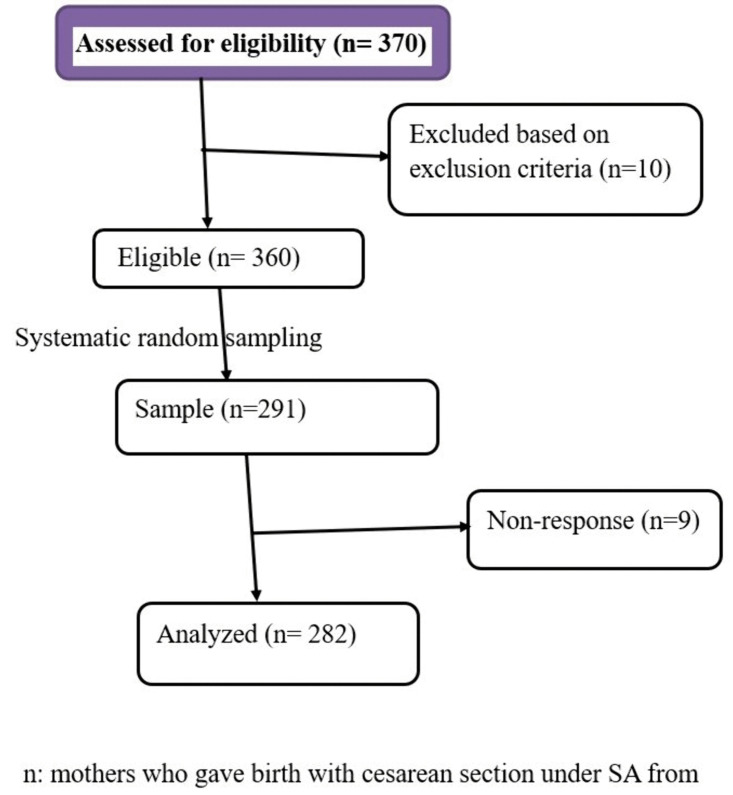
Flow diagram of selection for study on the prevalence and associated factors of PDPH in patients undergoing cesarean sections following SA at HFCSUH.

All women who gave birth with a cesarean section under SA at HFCSUH in Eastern Ethiopia were the source population, while all systematically randomly selected women who gave birth with a cesarean section under SA from November 20/2024, to January 20/2025, at HFCSUH in Eastern Ethiopia were the study population and included in the study. Women who needed general anesthesia due to failed SA during the procedure, had complications like active bleeding, were excluded from this study. The sample size for the first objective was calculated using the single population proportion formula. The sample size for the second objective (associated factors of post-dural puncture headache) was calculated by considering the double population proportion formula. The largest sample size is found to be **291** from the first objective by considering the assumptions: 95% confidence level, 80% power, and the margin of error between the sample and the population (0.05). The prevalence of PDPH is 20.2 [24], and 10% of non-respondents; therefore, it was taken as the final sample size. A systematic random sampling technique was employed to select the study population. The sampling frame was prepared using the women’s medical registration numbers to adjust the Kth value based on sample size determination and the source of the population. Then, the “K” value was calculated by dividing the total population of cesarean section patients (360) by the calculated sample size (291). Which means, K = N/n, 360/291 = 1.23, so we took K = 2 by rounding the decimal in order to randomly select the first participant from options 1 and 2 using a lottery method. Then, we added 1.23 to determine the second participant and applied standard mathematical rounding. Our study was purely observational and did not require prospective registration in a clinical trial. A total of 291 were sampled, and 9(3.09%) were non-responders. Finally, 282 study participants were interviewed (Fig 1).

### Data collection and quality control

Data were collected through face-to-face interviews with patients using a structured and pretested questionnaire adapted from previous studies with some modification, including adding clinical factors such as comorbidities [13,18]. Data regarding patients’ conditions were also taken from their medical records. The tools consisted of four parts: Section One covers patient identification, Section Two addresses patients’ socio-demographic characteristics, Section Three focuses on the anesthetic and clinical characteristics of patients, and Section Four provides follow-up data for patients (S1 Appendix). The questionnaire was translated into the local languages (Amharic and Afan Oromo) in written form and was translated back to the English version after data collection for its analysis and processing. A pre-test was conducted on 5% of of the sample size at another hospital (Jugal General Hospital). For data collection, one anesthesia resident and one BSc midwifery student were assigned as data collectors, and one anesthesia resident as a supervisor was recruited. The principal investigator trained the data collectors before the data collection began to avoid any preventable sources of error. The principal investigator and one resident supervised the overall data collection process so that the data collectors could report any issues during the data collection period.

### Variables

The primary outcome of this study was the prevalence of PDPH

### Operational definitions

**PDPH:** described as the headache after a lumbar puncture. The headache either occurs or intensifies within 15 minutes of standing up and subsides or improves within 30 minutes of lying back down [25].

### PDPH Headache Severity [26]

I. **Mild headache:** numerical pain score (1-3) with no limitation of activity, and no treatment required
II. **Moderate headache:** numerical pain score (4-6) with limited activity, and regular analgesics may be required
III. **Severe headache:** numerical pain score (7-10) with the patient confined to bed; anorexic

**Body Mass Index (BMI)**: The BMI of the patient was computed by taking the patient’s weight and height and was calculated by dividing the weight of patient’s weight in kilograms (kg) by the square height of the patient in meters (m^2^).

**Spinal Anesthesia:** injection of a local anesthetic into the CSF in the spinal canal to block sensory and motor sensations before they reach the central nervous system. Spinal needles ranging from 22G to 27G. The most commonly used type of needle for SA was cutting needles in the study area.

**Non-responders** are individuals or entities that do not participate in a survey or study after being invited or selected.

A **large spinal needle** is ≤ 23 gauge.

**Neck stiffness:** correlate self-reported measures of neck stiffness with objective assessments conducted by clinicians in the same context. It is the experience of pain and limited movement reported by the patient, and limited range of motion or tightness in the neck muscles during a physical examination.

### Data processing and analysis

Responses in each question were coded for simplicity of data entry. The coded data were entered into EpiData 4.6 and exported to SPSS version 20 statistical software for data analysis. The collected data were checked for consistency and completeness. In the first step, a descriptive analysis, such as percentages, frequency distribution, and measures of central tendency, was computed. Statistical analysis was conducted using the Chi-square test. Binary logistic regression was done, and variables were selected a priori based on established biological plausibility and previous evidence cited in the introduction. A multivariable logistic regression was performed to identify the independent predictors of PDPH and to control the effect of potential confounding variables using adjusted odds ratios with their corresponding 95% confidence intervals. Statistically significant level was declared at a p-value of less than 0.05. Multicollinearity and the fitness of the model were checked. Model fitness was tested by the Hosmer-Lemeshow goodness-of-fit statistics. Missing data were minimal and handled using the Listwise deletion method.

## Results

### Socio-demographic characteristics of study participants

A total of 282 study participants were interviewed from HFCSUH in Eastern Ethiopia with a response rate of 97% (Fig 1). The mean age of the respondents was 26.85 (±6.03SD) years, and 211 (74.8%) were aged between 18 and 30 years. More than half (53.2%) of the respondents were urban residents, and 255 (65.7%) of them had a BMI of between 18.00-24.9 kg/m^2^ (Table 1).

**Table 1:** Socio-demographic characteristics of the study participants in HFCSUH, Eastern Ethiopia, from November 20/2024-January 20/2025.

Variables	Category	Frequency (N)	Percentage (%)
Age	≤17 18-30 ≥31	11 211 60	3.9 74.8 21.3
Residency	Urban Rural	150 132	53.2 46.8
BMI (kg/m^2^)	<18	6	2
	18.00-24.9	77	27.3
	25.0-29.90	179	63.6
	≥30	20	7.1
Parity	≤3 >3	152 130	53.9 46.1

### Anesthetic characteristics of study participants

A similar cutting needle was used in all participants, and a ≤ 23-gauge needle (large spinal needle) was used in 70.9% of them. Most cesarean sections (78%) were done by emergency cesarean sections. Almost all patients (99.3%) had been given SA in a sitting position. SA performed with the needle orientation concerning the long axis of the spine was parallel in 235(83.3%) of the respondents. More than half of the respondents (53.5%) reported that anesthetists needed to attempt twice to succeed for the planned procedure. More than two-thirds (81.6%) of the procedures were performed by anesthesia practitioners who had greater than two years of working experience (Table 2).

**Table 2:** Anesthetic characteristics of the study participants.

Variables	Category	Frequency (N)	Percentage (%)
Needle size	≤ 23 >23	200 82	70.9 29.1
Position	Sitting Lateral	280 2	99.3 0.7
Number of attempts	>2 attempts Twice attempt Single attempt	86 151 45	30.5 53.5 16
Type of cesarean section	Elective Emergency	62 220	22 78
Approach to spinal anesthesia	Midline Paramedian	280 2	99.3 0.7
Orientation of the bevel	Parallel Perpendicular	235 47	83.3 16.7
Time of experienced staff in years	<2 ≥2	52 230	18.4 81.6

### Prevalence of PDPH and clinical characteristics of study participants

The majority of the participants (92.2%) didn’t have any comorbidities, and (98.6%) didn’t have preexisting headaches. More than half of the respondents (56.4%) had a previous history of SA, and 150 (53.2%) respondents explained a previous history of PDPH. In this study, PDPH was observed in 110 patients (39%, 95% CI: 33–45%). Of 110 patients who developed PDPH, more than half, 61(55.5%), developed after 24 hours but within 48 hours of the procedure. Among 110 patients who developed PDPH, 40 patients developed neck stiffness. Fifty patients (45.5%) developed moderate pain, followed by mild pain in 48(43.6%). Patients who experienced PDPH were treated using oral fluids (38.2%) and analgesics (30%) (Table 3).

**Table 3:** Clinical characteristics of the study participants.

Variables	Category	Frequency (N)	Percentage (%)
Comorbidities	Yes No	22 260	7.8 92.2
Preexisting headache	Yes No	4 278	1.4 98.6
Previous spinal anesthesia	Yes No	159 123	56.4 43.6
Previous PDPH	Yes No	150 132	53.2 46.8
Onset of time	Within the first 24 hours Within 24–48 hours Within 48–72 hours After 3 days, within 14 days	23 61 14 12	20.9 55.5 12.7 10.9
Severity of headache	Mild Moderate Severe	48 50 12	43.6 45.5 10.9
Associated sign and symptoms	Neck stiffness Low back pain Nausea Vomiting Vertigo Tinnitus	40 31 10 8 12 9	36.4 28.2 9 7.3 10.9 8.2
Measure	Take rest Fluid diet Iv fluid Caffeine Analgesia	17 42 12 6 33	15.5 38.2 10.9 5.4 30

### Factors associated with PDPH

The following variables were selected on clinical and theoretical grounds: spinal needle size, needle orientations, type of cesarean section, prior history of SA, previous history of PDPH, and BMI. In the multivariable logistic analysis, previous history of SA, previous history of PDPH, spinal needle size, and types of cesarean section were significantly associated with PDPH at a p-value of less than 0.05.

In this study, patients who had a previous history of PDPH were more likely to develop PDPH than their counterparts (AOR = 1.87; 95% CI: 1.08-3.27). Respondents who had a previous history of spinal anesthesia had higher odds of developing PDPH than their counterparts (AOR = 2.05; 95% CI: 1.16-3.65). The use of a large spinal needle size had increased odds of developing PDPH than a small size needle (AOR = 2.25; 95% CI: 1.22-4.16). Patients who had emergency cesarean sections were more likely to develop PDPH than their counterparts (AOR = 2.72; 95% CI: 1.28-5.80) (Table 4).

**Table 4:** Factors associated with PDPH of the study participants.

Variable	Category	PDPH(N = 282)		COR (95% Cl)	AOR (95% Cl)	P-value
		Yes n (%)	No n (%)			
Previous spinal anesthesia	No Yes	35(12.4) 75(26.6)	88(31.2) 84(29.8)	1 2.24(1.361-3.703)	1 2.05(1.155-3.646)	**0.014** ^*^
Previous PDPH	No Yes	38(13.5) 72(25.5)	94(33.3) 78(27.7)	1 2.28(1.393-3.744)	1 1.87(1.081-3.246)	**0.025** ^*^
BMI (kg/m^2^)	<18 18.0-24.9 25.0-29.90	2(0.8) 35(12.4) 66(23.4)	4(1.65) 43(15.2) 112(39.7)	1 0.61(0.106-3.533) 0.85(.151-4.759)	1 0.64(0.097-4.238) 1.11 (0.177-6.992)	0.643 0.910
	≥30	7(2.5)	13(4.6)	0.93(.135-6.398)	0.69(0.083-5.670)	0.728
Needle size	>23 ≤23	22(7.8) 88(31.2)	60(21.3) 112(39.7)	1 2.14(1.221-3.761)	1 2.25(1.223-4.135)	**0.009** ^*^
Type of cesarean section	Elective Emergency	11(3.9) 99(35.1)	51(18.1) 121(42.9)	1 3.79(1.877-7.667)	1 2.72(1.276-5.807)	**0.010** ^*^
Needle orientations	perpendicular parallel	14(5) 96(34)	33(11.7) 139(49.3)	1 1.63(0.827-3.204)	1 1.86(0.884-3.902)	0.102

*Significant variable at a p-value< 0.05, PDPH, Post Dural Puncture Headache

## Discussions

This study was done to assess the prevalence and associated factors of PDPH in cesarean section patients following SA at HFCSUH, Harar, Eastern Ethiopia. In this study, the overall prevalence of PDPH was observed to be more than one-third among the study population. The findings of this study identified previous history of SA, previous history of PDPH, large spinal needle size, and emergency cesarean sections as independent predictors of PDPH.

The prevalence of PDPH in this study was in line with other studies that were conducted at the University of Gondar Teaching and Referral Hospital, Gondar, Ethiopia [13]. The prevalence of PDPH in this study was higher than studies conducted in Jordan [11], Nigeria [12], Turkey [27], and Debre Tebor, Ethiopia [24]. Study method, population, exclusive use of cutting needles, and clinical setup differences may be the potential reasons for this difference. In our study, 99.3% of procedures were performed in the sitting position, and 84% of respondents needed to attempt twice or more to succeed at the planned procedure, compared to a study conducted at Debre Tebor Hospital, where 93% were in the sitting position, and 62.2% of procedures were attempted. However, these study findings were lower than those of other studies that were conducted in Somaliland [28]. The discrepancy may be attributed to variations in clinical setups differences and sociodemographic characteristics.

This study revealed that patients who had a previous history of SA were more likely to develop PDPH. This finding agrees with another study that was conducted at Debre Tabor General Hospital, Ethiopia [24]. This could be because a previous dural puncture might result in a weakened or altered dural structure, increasing the prevalence of PDPH with repeated dural puncture [29]. Similarly, patients who had a previous history of PDPH were more likely to develop PDPH in this study, which aligns with other studies conducted in the comprehensive specialized Referral Hospital of Northwest Ethiopia [18] and Brazil [30]. The association might be explained by the fact that PDPH increases the brain’s sensitivity to changes in CSF pressure, and having a history of these headaches increases the likelihood of getting another one [31].

According to the findings of this study, a significant association was observed between large spinal needle size and PDPH. Patients who received SA using bigger spinal needles (< 23-gauge) were more likely to develop PDPH than patients who received SA using smaller needles. This is in line with the studies conducted at Debre Tabor General Hospital, Ethiopia [24], and at Wolaita Sodo University, Ethiopia [32]. This might be because larger holes allow more CSF to leak, making it less likely to heal on its own [33]. However, there is no association with the outcome variable regarding the type of design of the needle, as all were cutting types.

The findings of this study showed that respondents who had emergency cesarean sections were more likely to develop PDPH than those who had cesarean sections performed electively. This is similar to the findings from a previous study [34]. Emergency cesarean sections may require more attempts at SA, and there may be less time to optimally position the patient for SA, which increases the risk of dural puncture and subsequent CSF leakage, and this leakage can cause headaches [35].

Provider inexperience was not found to be significant in our study. However, another study has shown that provider inexperience is a risk factor for PDPH [15]. A study conducted in Gedeo Zone hospitals found that SA performed by an anesthetist with more than 3 years of experience reduced the incidence of PDPH by 56% when compared to an anesthetist with less than 3 years of experience [36]. It has been demonstrated that more meningeal punctures, which are often linked to inexperience, have been shown to increase the rate of PDPH [37].

Studies have shown that the use of cutting needles is associated with a higher prevalence of PDPH [38]. However, cutting needles might have been more readily available in Ethiopia [39]. Cutting needles have a sharp point and a cutting bevel that create a puncture hole and a perforation channel through the dura mater [40].

### Limitations of the study

The study has some limitations. Some participants had difficulties remembering details of previous events, which may affect the precision of the findings. Temporality and causal inferences could not be established because of the nature of the cross-sectional study design. In addition, the study was limited to one hospital (HFCSUH) and cannot generalized to other hospitals in the Harari region. This affects its external validation.

## Conclusion

The prevalence of PDPH in this study was higher than in most other studies. Factors such as previous history of SA, previous history of PDPH, patients who received SA using bigger spinal needles, and respondents who had emergency cesarean sections were found to be significantly associated with PDPH. The findings of this study suggest that the healthcare providers should pay special attention to emergency cesarean deliveries for women with a previous history of SA and PDPH, as well as providing smaller-gauge spinal needles and pencil-point needles, which would be beneficial in reducing PDPH in patients undergoing cesarean sections.

Additionally, researchers are encouraged to conduct prospective cohort studies with multicenter and large sample sizes in the future.

## Supporting information

S1 Dataset This is the data set of a study of prevalence and associated factors of PDPH in cesarean section patients following SA. (XLSX)

S1 Appendix This appendix contains the consent form and questionnaire for the study on prevalence and associated factors of PDPH in cesarean section patients following SA. (DOCX)

## References

[1] Dabas R, Lim MJ, Sng BL. Postdural puncture headache in obstetric neuraxial anaesthesia: current evidence and therapy. Trend Anaesthesia Critical Care. 2019;25:4–11. doi: 10.1016/j.tacc.2019.01.002

[2] Sachs A, Smiley R. Post-dural puncture headache: the worst common complication in obstetric anesthesia. Semin Perinatol. 2014;38(6):386–94. doi: 10.1053/j.semperi.2014.07.007 25146108

[3] Guglielminotti J, Landau R, Li G. Major neurologic complications associated with postdural puncture headache in obstetrics: a retrospective cohort study. Anesth Analg. 2019;129(5):1328–36. doi: 10.1213/ANE.0000000000004336 31335402 PMC9924132

[4] Sury MRJ, Palmer JHMG, Cook TM, Pandit JJ. The state of UK anaesthesia: a survey of National Health Service activity in 2013. Br J Anaesth. 2014;113(4):575–84. doi: 10.1093/bja/aeu292 25236896

[5] Kassa MW, Mkubwa JJ, Shifa JZ, Agizew TB. Type of anaesthesia for caesarean section and failure rate in Princess Marina Hospital, Botswana’s largest referral hospital. Afr Health Sci. 2020;20(3):1229–36. doi: 10.4314/ahs.v20i3.26 33402969 PMC7751529

[6] Aregawi A, Terefe T, Admasu W, Akalu L. Comparing the effect of spinal and general anaesthesia for pre-eclamptic mothers who underwent caesarean delivery in A tertiary, Addis Ababa, Ethiopia. Ethiop J Health Sci. 2018;28(4):443–50. doi: 10.4314/ejhs.v28i4.10 30607057 PMC6308727

[7] Plewa MC, Hall WA, McAllister RK. Postdural puncture headache. StatPearls. Treasure Island (FL): StatPearls Publishing; 2025. 28613675

[8] Headache Classification Committee of the International Headache Society (IHS) The International Classification of Headache Disorders, 3rd edition. Cephalalgia. 2018;38(1):1–211. doi: 10.1177/0333102417738202 29368949

[9] Ljubisavljevic S. Postdural puncture headache as a complication of lumbar puncture: clinical manifestations, pathophysiology, and treatment. Neurol Sci. 2020;41(12):3563–8. doi: 10.1007/s10072-020-04757-z 32997283

[10] Chekol B, Yetneberk T, Teshome D. Prevalence and associated factors of post dural puncture headache among parturients who underwent cesarean section with spinal anesthesia: a systemic review and meta-analysis, 2021. Ann Med Surg (Lond). 2021;66:102456. doi: 10.1016/j.amsu.2021.102456 34141426 PMC8187936

[11] Khraise WN, Allouh MZ, El-Radaideh KM, Said RS, Al-Rusan AM. Assessment of risk factors for postdural puncture headache in women undergoing cesarean delivery in Jordan: a retrospective analytical study. Local Reg Anesth. 2017;10:9–13. doi: 10.2147/LRA.S129811 28360535 PMC5364012

[12] Ayyuba R, Mohammed A, Salisu I, Nagoma A, Owolabi L, Ibrahim A. An analysis of postdural puncture headache in obstetric patients: a study from Kano, Nigeria. Trop J Obstet Gynaecol. 2017;34(1):16. doi: 10.4103/tjog.tjog_61_16

[13] Kassa AA, Beyen TK, Denu ZA. Post dural puncture headache (PDPH) and associated factors after spinal anesthesia among patients in University of Gondar Referral and Teaching Hospital, Gondar, North West Ethiopia. Trop J Obstetrics Gynaecol. 2015;34(1). doi: 10.4314/tjog.v34i1

[14] Kwak K-H. Postdural puncture headache. Korean J Anesthesiol. 2017;70(2):136–43. doi: 10.4097/kjae.2017.70.2.136 28367283 PMC5370299

[15] Uppal V, Russell R, Sondekoppam R, Ansari J, Baber Z, Chen Y, et al. Consensus practice guidelines on postdural puncture headache from a multisociety, international working group: a summary report. JAMA Netw Open. 2023;6(8):e2325387. doi: 10.1001/jamanetworkopen.2023.25387 37581893

[16] Davoudi M, Tarbiat M, Ebadian MR, Hajian P. Effect of position during spinal anesthesia on postdural puncture headache after cesarean section: a prospective, single-blind randomized clinical trial. Anesth Pain Med. 2016;6(4):e35486. doi: 10.5812/aapm.35486 27843773 PMC5100205

[17] Ayub F, Ahmad A, Aslam KZ, Saleem I. Frequency of headache with 25G or 27G quincke needles after spinal anesthesia in patients undergoing elective cesarean section. Anaesthesia, Pain Intensive Care. 2019;:170–3.

[18] Mekete G, Demelash H, Almaw A, Seid S. Magnitude and associated factors of post Dural puncture headache after spinal anesthesia in surgical patients at comprehensive specialized referral hospital, 2021: a multi-center cross-sectional study. Interdiscip Neurosurg. 2023;34:101817. doi: 10.1016/j.inat.2023.101817

[19] Peralta F, Higgins N, Lange E, Wong CA, McCarthy RJ. The relationship of body mass index with the incidence of postdural puncture headache in parturients. Anesth Analg. 2015;121(2):451–6. doi: 10.1213/ANE.0000000000000802 25993388

[20] Arevalo-Rodriguez I, Muñoz L, Godoy-Casasbuenas N, Ciapponi A, Arevalo JJ, Boogaard S, et al. Needle gauge and tip designs for preventing post-dural puncture headache (PDPH). Cochrane Database Syst Rev. 2017;4(4):CD010807. doi: 10.1002/14651858.CD010807.pub2 28388808 PMC6478120

[21] Naghibi K, Hamidi M. Prophylactic administration of aminophylline plus dexamethasone reduces post-dural puncture headache better than using either drug alone in patients undergoing lower extremity surgery. Adv Biomed Res. 2014;3:5. doi: 10.4103/2277-9175.124631 24600595 PMC3929052

[22] Halker RB, Demaerschalk BM, Wellik KE, Wingerchuk DM, Rubin DI, Crum BA, et al. Caffeine for the prevention and treatment of postdural puncture headache: debunking the myth. Neurologist. 2007;13(5):323–7. doi: 10.1097/NRL.0b013e318145480f 17848873

[23] Hadavi SMR, Panah A, Shamohammadi S, Kanaani Nejad F, Sahmeddini MA, Asmarian N. The prophylactic effect of acetaminophen and caffeine on post dural puncture headache after spinal anesthesia for cesarean section: a randomized double-blind clinical trial. Iran J Med Sci. 2024;49(9):573–9. doi: 10.30476/ijms.2023.99577.3166 39371383 PMC11452584

[24] Demilew BC, Tesfaw A, Tefera A, Getnet B, Essa K, Aemero A. Incidence and associated factors of postdural puncture headache for parturients who underwent cesarean section with spinal anesthesia at Debre Tabor General Hospital, Ethiopia; 2019. SAGE Open Med. 2021;9:20503121211051926. doi: 10.1177/20503121211051926 34676076 PMC8524678

[25] Vallejo MC, Zakowski MI. Post-dural puncture headache diagnosis and management. Best Pract Res Clin Anaesthesiol. 2022;36(1):179–89. doi: 10.1016/j.bpa.2022.01.002 35659954

[26] Bielewicz J, Daniluk B, Kamieniak P. VAS and NRS, same or different? Are visual analog scale values and numerical rating scale equally viable tools for assessing patients after microdiscectomy?. Pain Res Manag. 2022;2022(1):5337483. doi: 10.1155/2022/5337483 35391853 PMC8983264

[27] Pirbudak L, Özcan HI, Tümtürk P. Postdural puncture headache: Incidence and predisposing factors in a university hospital. Agri. 2019;31(1):1–8. doi: 10.5505/agri.2018.43925 30633317

[28] Mohamed G, Sukar KAO, Guled ESH, Elamin SMA, Kandakurti PK, Fathelrahman M, et al. Multiple risk factors analysis of post-dural puncture headache (PDPH) among parturient patients underwent caesarean section at obstetric hospitals in Somaliland. The Open Anesthesia J. 2024;18(1). doi: 10.2174/0125896458339013241009102454

[29] Jabbari A, Alijanpour E, Mir M, Bani Hashem N, Rabiea SM, Rupani MA. Post spinal puncture headache, an old problem and new concepts: review of articles about predisposing factors. Caspian J Intern Med. 2013;4(1):595–602. 24009943 PMC3762227

[30] Amorim JA, Gomes de Barros MV, Valença MM. Post-dural (post-lumbar) puncture headache: risk factors and clinical features. Cephalalgia. 2012;32(12):916–23. doi: 10.1177/0333102412453951 22843225

[31] Shahriari A, Sheikh M. Post-spinal headache: a new possible pathophysiology. Anesth Pain Med. 2016;7(1):e42605. doi: 10.5812/aapm.426056 28920045 PMC5554423

[32] Weji BG, Obsa MS, Melese KG, Azeze GA. Incidence and risk factors of postdural puncture headache: prospective cohort study design. Perioper Med (Lond). 2020;9(1):32. doi: 10.1186/s13741-020-00164-2 33292510 PMC7650180

[33] Halpern S, Preston R. Postdural puncture headache and spinal needle design. Metaanalyses. Anesthesiology. 1994;81(6):1376–83. doi: 10.1097/00000542-199412000-00012 7992906

[34] Kambale M, Jadhav SJ. Incidence of post-dural lumbar puncture headache (PDLPH) in comparison between emergency and elective lower segment cesarean section (LSCS) with 26G Quincke-Babcock cutting-beveled spinal needle. Saudi J Anaesth. 2024;18(3):338–45. doi: 10.4103/sja.sja_950_23 39149748 PMC11323915

[35] Thakur S, Sharma A, Kaushal S, Sharma A, Sharma N, Thakur PS. Incidence and risk factors of “postdural puncture headache” in women undergoing cesarean delivery under spinal anesthesia with 26g quincke spinal needle, experience of medical college in rural settings in India 2019: a prospective cohort study design. J Pharm Bioallied Sci. 2022;14(Suppl 1):S209–13. doi: 10.4103/jpbs.jpbs_72_22 36110769 PMC9469433

[36] Girma T, Mergia G, Tadesse M, Assen S. Incidence and associated factors of post dural puncture headache in cesarean section done under spinal anesthesia 2021 institutional based prospective single-armed cohort study. Ann Med Surg (Lond). 2022;78:103729. doi: 10.1016/j.amsu.2022.103729 35600186 PMC9121279

[37] Kim JE, Kim SH, Han RJW, Kang MH, Kim JH. Postdural puncture headache related to procedure: incidence and risk factors after neuraxial anesthesia and spinal procedures. Pain Med. 2021;22(6):1420–5. doi: 10.1093/pm/pnaa437 33675230

[38] Lee SI, Sandhu S, Djulbegovic B, Mhaskar RS. Impact of spinal needle type on postdural puncture headache among women undergoing Cesarean section surgery under spinal anesthesia: a meta-analysis. J Evid Based Med. 2018;11(3):136–44. doi: 10.1111/jebm.12311 30070060

[39] Bayable SD, Ahmed SA, Lema GF, Yaregal Melesse D. Assessment of maternal satisfaction and associated factors among parturients who underwent cesarean delivery under spinal anesthesia at university of gondar comprehensive specialized hospital, Northwest Ethiopia, 2019. Anesthesiol Res Pract. 2020;2020(1):8697651. doi: 10.1155/2020/8697651 33101405 PMC7576364

[40] Siddiqui AS, Salim B, Hashemy N, Siddiqui SZ. Post-dural puncture headache after spinal anaesthesia for caesarean section. J Surgery Pakistan (International). 2015;20(1):1.

