# Supplementary material for: Prevalence and associated factors of postdural puncture headache in cesarean section patients following spinal anesthesia in a public hospital in Harar, Eastern Ethiopia
Source: PLOS Glob Public Health. 2026 Jun 2;6(6):e0006581. doi: 10.1371/journal.pgph.0006581 (PMC13229333; doi:10.1371/journal.pgph.0006581)
Supplement: S1 Appendix — (DOCX) [file pgph.0006581.s002.docx]

# APPENDIX

## Information Sheet and Informed Voluntary Consent Form for Study Participants

My name is____________________. I am working as a data collector for a study conducted by X, who is a graduating student at Haramaya University, College of Health and Medical Sciences, Department of Anesthesiology, Critical Care, and Pain Medicine. I kindly request you to lend me your attention to explain to you about the study and the study participants.

**The study title:** Prevalence and associated factors of postdural puncture headache in cesarean section patients following spinal anesthesia at Hiwot Fana Comprehensive Specialized Hospital, Harar, Eastern Ethiopia.

**Purpose of the study:** The main aim of this study is to assess the prevalence and associated factors of postdural puncture headache in cesarean section patients following spinal anesthesia at Hiwot Fana Comprehensive Specialized Hospital, Harar, Eastern Ethiopia. Moreover, the result of the study will help to improve the health services provided by health facilities.

**Procedure and duration:** I will interview the participants using a questionnaire with 31 questions to provide me with pertinent data that is helpful for the study. The interview will take about 20 minutes.

**Risk and benefits:** The risk of participating in this study is minimal. But a few minutes from the participants' time. There would not be any direct payment for reviewing in this study. But, the findings from this research will reveal important information for the healthcare giver and for local healthcare planners.

**Confidentiality:** The information that we will be provided will be kept confidential. There will be no information that will identify the participant in particular. The findings of the study will be general for the study participants and will not reflect anything particular about a person. The questionnaire will be coded to exclude showing names. No reference in oral or written reports that could link the participant to research.

**Rights:** Participation in this study is fully voluntary. The participants have the right to declare whether to participate or not in this study. If they decide to participate, they have the right to withdraw from the study at any time, and this will not label them for any loss of benefits which they otherwise are entitled to. They do not have to answer any question that they do not want to answer.

**Contact address:** If there are any questions or inquiries at any time about the study or procedures, please contact this address. Institutional Health Research Ethics Review Committee; Office phone: +251-254-66-2011

P.O. Box 235, Harar, Ethiopia.

**Declaration of informed voluntary consent**:

I have read this form, or it has been read to me in the language I understand. I have clearly understood the purpose of research, the procedures, the risks and benefits, issues of confidentiality, the rights of participating, and the contact address for any queries. I have been allowed to ask questions about things that may have been unclear. I was informed that I have the right to withdraw from the study at any time or not to answer any question that I do not want to. Therefore, I declare my voluntary consent to participate in this study with my signature as indicated below.

Name and signature of participant ____________________________ Date _________

Name and signature of data collector__________________________________ Date _________

**Thank you for your cooperation!!**

## Questionnaire

**Part one: Patient Identification**

| Ser. No | Items | Responses |
| --- | --- | --- |
| 101 | Serial number | _____________ |
| 102 | Date of admission | _____________ |
| 103 | Contact Telephone number | ____________ |

**Part two: Sociodemographic characteristics**

| 201 | Age in years | __________ |
| --- | --- | --- |
| 202 | Parity | __________ |
| 203 | Residency | a) Urban  b) Rural |
| 204 | Height in meter | __________ |
| 205 | Weight in Kg | __________ |
| 206 | Previous history of spinal anesthesia | a) Yes  b) No |
| 207 | Previous history of PDPH | a) Yes  b) No |

**Part three: Anesthetic and clinical characteristics**

| 301 | The time at which the spinal anesthesia is given | __________ |
| --- | --- | --- |
| 302 | Spinal needle used | a) Type (design)_______  b) Size_______________ |
| 303 | Position | a) Siting  b) Lateral |
| 304 | Number of attempts | a) Single attempt  b) Twice attempt  c) >2 attempts |
| 305 | Type of cesarean section | a) Emergency  b) Elective |
| 306 | Approach to spinal anesthesia | a) Midline  b) Paramedian |
| 307 | Orientation of the needle bevel to the long axis of the spine | a) Parallel  b) Perpendicular |
| 308 | Provider’s experience in years | -----------------year |
| 309 | Preexisting headache | a. yes  b. no |
| 310 | Comorbidities  (If yes, go to next question) | a. yes  b. no |
| 311 | Diabetes mellitus | a. yes  b. no |
| 312 | Cardiovascular disease | a. yes  b. no |
| 313 | Stroke | a. yes  b. no |
| 314 | Kidney disease | a. yes  b. no |
| 315 | Asthma | a. yes  b. no |
| 316 | Others | a. yes  b. no |

**Part four: Follow-up Data in Maternity Wards**

| 401 | PDPH | a) Present  b) Absent |
| --- | --- | --- |
| 402 | Time of onset of PDPH after spinal block | a) Within the 1st 24hrs.  b) After 24hrs within 48hrs.  c) After 48hrs within 72hrs.  d) After 3 days, within 14 days |
| 403 | Severity of headache | a) Mild  b) Moderate  c) Severe |
| 404 | Other symptoms associated with PDPH | a) Neck stiffness  b) Low back pain  c) Nausea  d) Vomiting  e) Vertigo  f) Tinnitus |
| 405 | What measures do you take for your headache? | A) Take a rest  B) Fluid diet  C) IV fluid  D) Caffine(Coffee, Tea, Cola)  E) Analgesic   1. Paracetamol 2. Diclofenac 3. Ibuprofen 4. Opoids (specify)______ 5. Other (Specify)_______ |
